# Acute Upper Airway Obstruction Due to Massive Cervical Subcutaneous Emphysema: A Case Report

**DOI:** 10.7759/cureus.34420

**Published:** 2023-01-30

**Authors:** Rani Hammoud, Fatima Emam, Suzan Mohamed, Hassanin Abdulkarim

**Affiliations:** 1 Otolaryngology - Head and Neck Surgery, Hamad Medical Corporation, Doha, QAT; 2 Radiology, Hamad Medical Corporation, Doha, QAT

**Keywords:** subcutaneous emphysema, boerhaave syndrome, difficult airway management, spontaneous esophageal perforation, acute airway obstruction

## Abstract

With upper airway obstruction being an emergency, a high index of suspicion and proper and timely treatment planning are crucial to the patient’s life. Spontaneous esophageal perforation, also known as Boerhaave syndrome, has been observed to cause subcutaneous emphysema; however, airway compromise secondary to subcutaneous emphysema is extremely rare when there is no associated broncho-tracheal injury. Here, we present a case of esophageal perforation complicated with cervical emphysema that led to acute airway obstruction requiring invasive ventilation.

## Introduction

Stabilization of the airway and respiratory and circulatory systems are the most important steps when evaluating a patient’s general condition. Proper airway management should be prompt and efficient. Otolaryngologists are frequently consulted to assist in airway management. Common causes of upper airway obstruction include infections, inflammation, trauma, mechanical obstruction, and iatrogenic diseases, but, rarely, subcutaneous emphysema [1]. Subcutaneous emphysema is usually a self-limiting condition that involves trapped air along the tissue planes [2]. However, if it extensively involves the hypopharynx, it can eventually lead to upper airway obstruction which can be fatal [3,4]. Fasciocervical subcutaneous emphysema can be primary or secondary to other etiologies, such as dental procedures, lung disease, infections, trauma, and esophageal perforation [2,3,5-7].

Here, we present a case of upper airway obstruction secondary to Boerhaave syndrome in a middle-aged Indian man without systemic disease.

## Case presentation

A 43-year-old Indian gentleman not known to have any comorbidities presented to the emergency department complaining of sudden, increasing neck swelling following a sensation of a foreign body stuck in the throat. The patient’s complaint was associated with a mild change in voice, difficulty swallowing solid food, multiple episodes of retching but no vomiting, and mild breathing difficulty. The patient had no chest pain, fever, hematemesis, or abdominal pain. The patient had no history of smoking, alcohol consumption, neck trauma, or ingestion of corrosive agents. However, he mentioned the need to drink water when eating meals since childhood but never sought medical advice.

**Figure 1:**
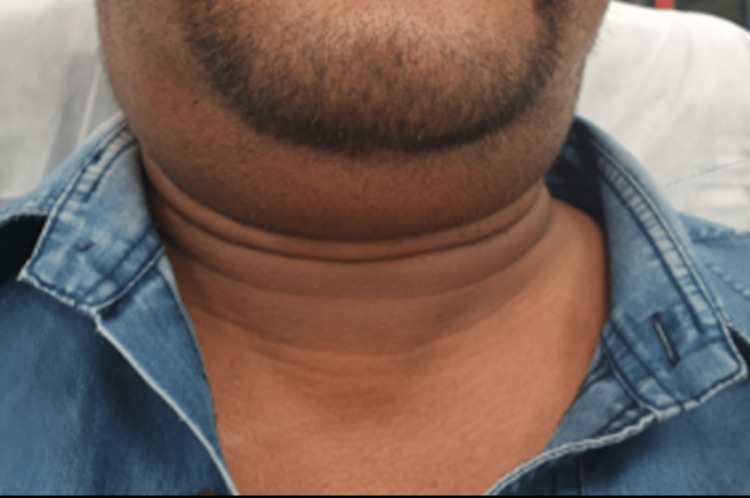
Anterior neck image showing the neck swelling on initial presentation.

General examination showed a slightly tachypneic adult maintaining oxygen saturation at room air, with no stridor or noisy breathing. Upon greeting the patient, a high-pitch vibratory and electronic-like voice was noticed as the patient was speaking in full sentences without distress. On palpation, he had a right-sided, non-tender neck swelling with crepitation (Figure 1).

Flexible fiberoptic examination revealed diffuse pharyngeal and supraglottic swelling that vibrated with speech and respiration, along with narrowing of the upper airway.

**Figure 2:**
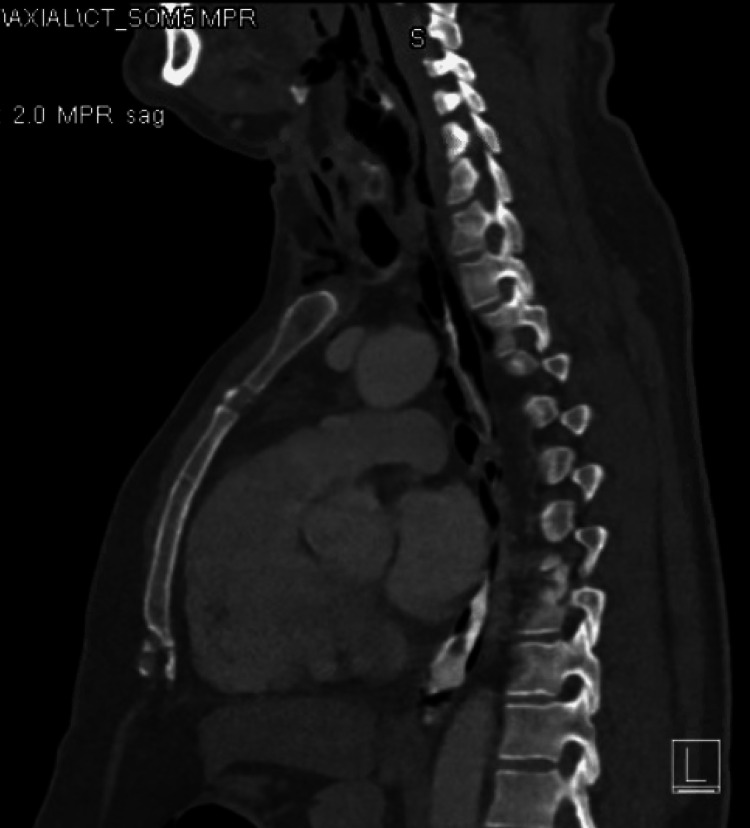
Sagittal CT imaging of the neck and chest.

CT of the neck and chest revealed intramural air involving the hypopharynx, esophagus, and stomach, associated with soft-tissue emphysema involving the neck with pneumomediastinum. Findings were highly suggestive of esophageal perforations at the pharyngeal-esophageal/proximal part of the esophagus (Figure 2).

**Figure 3:**
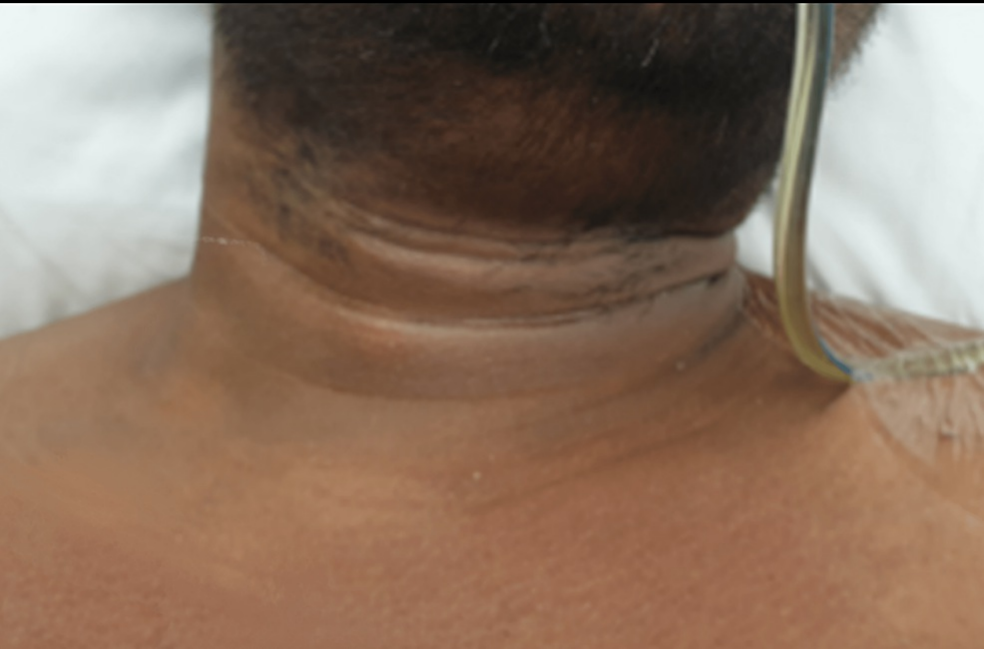
Anterior neck image showing resolution of the subcutaneous emphysema on day five.

The patient was transferred and monitored in the observational unit for any deterioration. As anticipated, briefly after his transfer, his symptoms exacerbated as he began to develop noisy breathing and respiratory distress. Therefore, the patient underwent a difficult emergency endotracheal intubation through a fiberoptic endoscope followed by an upper esophagoscope that showed an esophageal laceration 18 cm from the incisors. Otherwise, distal to the laceration, the esophagus was normal with no foreign body detected. Subsequently, the patient was shifted to the surgical intensive care unit while being maintained on ventilatory support. He was treated conservatively with prophylactic antibiotics, proton pump inhibitors for gastric acid suppression, nothing by mouth, and feeding through a nasogastric tube. After five days, the patient’s cervical emphysema improved (Figure 3), and he was successfully extubated without any complications. With the patient’s voice returning to normal, he was shifted to the regular floor on day seven. On day nine, he was started on a diet after a barium swallow excluded any remaining esophageal leak. On day 12, the patient was safely discharged home.

## Discussion

The term emphysema is of Greek origin that translates to swelling or inflation. In medical terminology, emphysema is used to describe air trapped inside a limited space in the human body [1]. Subcutaneous emphysema was first mentioned in the literature around the late 1910s [8]. It develops secondary to blunt or penetrating trauma to the chest, abdomen, neck, and sinus cavity, as well as following gastrointestinal perforation and barotrauma. In addition, some case reports have described subcutaneous emphysema after dental procedures [9], tonsillectomies [10], and laparoscopic surgery [11]. In our case, the patient developed subcutaneous emphysema following spontaneous esophageal rupture, also known as Boerhaave syndrome.

Boerhaave syndrome, first described by the German physician Herman Boerhaave in 1724 at Leiden university [12,13], is a spontaneous longitudinal transmural tear of the esophageal wall secondary to a sudden increase in the intraesophageal pressure that is usually followed by forceful vomiting. Boerhaave syndrome has been reported following weightlifting, seizures, and abdominal trauma [13,14]. It accounts for 15% of all cases of esophageal rupture [13]. The actual incidence of this condition is believed to be around 3.1/1,000,000 [13]. Although due to the high mortality of this condition which reaches up to 40%, a significant number of cases are diagnosed late, suggesting underreporting and underestimation of the incidence of Boerhaave syndrome [12,13,15].

Even though the clinical presentation of a patient with Boerhaave syndrome differs according to the location of the esophageal perforation, which tends to be most commonly at the distal part of the esophagus, 50% of cases present with Mackler’s triad of vomiting, lower thoracic pain, and subcutaneous emphysema [13]. In our case, we were contacted as otolaryngologists because the patient initially presented with non-specific symptoms suggestive of a foreign body sensation in his throat, which was later associated with increasing cervical emphysema and a change in voice. Data show that subcutaneous emphysema is observed in 28-66% of Boerhaave syndrome cases [16]. Although subcutaneous emphysema remains a rare finding with the overall general incidence ranging between 0.43% and 2.34% [17], airway compromise secondary to subcutaneous emphysema is extremely rare, let alone when there is no associated broncho-tracheal injury [18] because the subcutaneous air is easily accommodated by the flexible subcutaneous tissue. However, in our case, the patient’s rapidly worsening cervical emphysema and a change in his voice rendered the assessment of the upper airway a top priority. A flexible fiberoptic examination was, therefore, performed to evaluate the patency of the upper airway which revealed pending airway compromise. Arrangements were then made to prevent any delay in the case that the patient needed intubation or emergency tracheostomy.

Although esophageal perforation was top on our differential list, there was no history of sharp food consumption; therefore, causes other than traumatic esophageal perforation had to be considered. In the end, the patient was diagnosed with spontaneous perforation of the proximal esophagus complicated by upper airway obstruction. His airways were secured with endotracheal intubation, and the esophageal perforation was treated conservatively.

## Conclusions

Our case report emphasizes the importance of maintaining a high index of suspicion in the diagnosis of upper airway obstruction. Moreover, ongoing evaluation to prepare for potential airway compromise is imperative. Despite being rare, our patient developed an upper esophageal rupture that caused cervical subcutaneous emphysema, obstructing the previously patent upper airway.
